# ‘It is still coming from the centre and coming out’: The material conditions adding to over‐bureaucratised patient and public involvement for commissioning health and care in England

**DOI:** 10.1111/hex.13768

**Published:** 2023-04-26

**Authors:** Debbie Hatfield, Kay Aranda, Gordon Ferns, Breda Flaherty, Angie Hart

**Affiliations:** ^1^ School of Humanities and Social Science University of Brighton Brighton UK; ^2^ School of Sport and Health Sciences University of Brighton Brighton UK; ^3^ Department of Medical Education, Brighton and Sussex Medical School University of Brighton Brighton UK

**Keywords:** clinical commissioning, leadership, materiality, partnership, patient and public involvement (PPI), service users

## Abstract

**Objective:**

To understand how materiality affects patient and public involvement (PPI) for commissioning and leading health and care services in the English National Health Service (NHS) context.

**Context:**

From April 2013 groups of general practitioners (GPs) became members of NHS clinical commissioning groups (CCGs) to assess needs and procure core health services for and with local communities. Since July 2022, integrated care systems (ICSs) have subsumed this responsibility. NHS reorganisations have been driven by the promise of more effective and efficient health care and have led to a long history of PPI on economic, political, and moral grounds. Few studies researching PPI in clinical commissioning exist and fewer still have explored a more agentic understanding of materiality and its impact on PPI.

**Study Design:**

A focused ethnography was used to examine PPI for clinical commissioning within two CCG case study sites in England. Three CCG Governing Body lay representatives, nine GP commissioners and seven service user representatives took part in focus groups and/or were interviewed. Fifteen nonparticipant observations were also carried out at CCG meetings and the associated materiality was examined.

**Findings:**

The materiality of activities involved in clinical commissioning influences and shapes the nature of PPI. These forms of materiality may dilute and subvert meaningful engagement and involvement that relies on trust, leadership, learning, and partnership working.

**Conclusion:**

System leaders in ICSs should consider the significance of materiality in centrally driven processes involved in PPI commissioning to reduce barriers and ensure meaningful partnerships within local communities.

**Patient and Public Contribution:**

The study design ensured PPI throughout the research process in keeping with contemporary research practice guidance. The project steering committee included service users with current or recent PPI clinical commissioning experience outside of the study sites. There was PPI involvement in the original study proposal and its development including the bid for doctoral funds on which this study is based. All were involved in assessing the rigour of the data collection, interpretation of the findings and ensuring the project remained true to the aims of the study. Two members have also participated in presentation of the study findings.

## 1 BACKGROUND

At the time of the study, general practitioners (GPs) were taking on leadership roles for the commissioning of local secondary and community care services within geographical localities of over 200 new clinical commissioning groups (CCGs) in England, defined under the Health and Social Care Act 2012 (HSCA 2012). As statutory NHS membership organisations comprising local GP practices (general practices) CCGs were legally required to involve and engage service users (patients and carers) and the public in the commissioning of services, improving on their design and decommissioning where services were not efficient and effective. ‘Meaningful engagement with patients, carers and their communities’ was a requirement of the CCG authorisation process.^1^ Some CCGs had urban geographies aligned with local government jurisdiction whereas others were more rural, requiring patients to use hospital and secondary care services purchased from various providers in conurbations outside the CCG boundary.

Several iterations of NHS commissioning over the previous 20‐year period had culminated in further re‐organisation anticipating a greater role for GPs, and the public in the process,^2^, ^3^, ^4^ in addition to the Standard General Medical Services contract where general practices, as independent businesses, provide NHS primary care. The aim of this study was to explore how clinical commissioners, (predominantly GPs), members of the public and service users made significant decisions shaping local health and care services. It examined how clinicians and service users working together to commission and lead health services could inform and provide deeper understanding of partnership, public engagement, and clinical leadership. We have explored the overlooked materiality of patient and public involvement (PPI) in NHS clinical commissioning.

The ubiquitous nature of PPI potentially positions the patient as a consumer and contributor to shaping health and care services. The purchaser and provider split within UK health services from the 1990s furthered a shift to the political right and what Martin^5^ describes as a technocratic rationale for involving patients and the public. Lay expertise is seen as important for assessing value for money, choice and patient satisfaction. In contrast, a democratic rationale for PPI ^5^ is concerned with living life on more equal terms, providing a voice in public policy and health and welfare services to defend social rights and enhance representation.^6^

These different PPI imperatives have led to numerous debates about effective ways to make PPI more sustainable and relevant but even with research and best practice guides,^7^, ^8^, ^9^ the problems of tokenism and superficial engagement remain.^10^, ^11^ Most mitigations focus on the procedures refining practices, improving organisation, management and/or levels of education. Whilst some attention to the role of bureaucracy exists more generally, providing useful insights, very few studies have drawn on contemporary understandings of materiality involved in PPI and associated practices. We draw on recent sociomaterial theories where materiality is defined as materials, objects or things that are indivisible from meanings and social and cultural understandings, structuring experiences. These materials are more than mere backdrop, nor are they instrumental, inert or static. This understanding of materiality is entangled with the social, structuring lives, experiences and relations to others,^12^, ^13^ and suggests matter is agentic and significantly active.^10^

The importance of this account of materiality is evident in notions of participation as advocated by Wenger's^14^ ‘communities of practice’ (CoP) theory. Practices comprise materials and meaning which are always ongoing and incomplete. This is evident in, for example, specified criteria, regulations, symbols, documents and contracts as identified in Table 1.

**Table 1:** The social and material aspects of participation.

**Social**	**Material**
What is said/unsaid	Language
What is represented/assumed	Tools
Subtle cues	Documents
Untold rules of thumb	Images
Recognisable intuitions	Symbols
Well‐tuned sensitivities	Well‐defined roles
Specific perceptions	Specified criteria
Underlying assumptions	Codified procedures
Embodied understandings	Regulations
Shared world views	Contracts

*Source*: Adapted from Wenger.^14^

Similarly, Fenwick,^15^ differentiating between the material and social forces of practice learning, refers to the ‘everyday stuff of our lives’ including furniture, forms, checklists, minutes, and databases. Examples of such materiality^12^ indicate the material conditions for PPI since CCG governance required a particular way of reporting for transparency, even to the style of template used for minuting purposes. Thus, as Wenger observes, if both participation and reification (making practice concrete) are influenced by excessive processes and procedures, so too are the social meanings which reinforce policies and legislation.^14^

‘Excess bureaucracy’ and inefficient processes in the English health and care system have been acknowledged:

excessively complex rules (whether legal, organisational or cultural) or assurance and reporting administrative processes, which either have no benefit, or have no net benefit as they are unduly resource intensive, inefficient and time consuming. DHSC.^16^

Therefore, new legislation intends to reduce the bureaucracy, remove competition between purchasers and providers in the NHS and encourage more co‐operation and collaboration.^17^, ^18^, ^19^ The Health and Care Act 2022 saw 42 Integrated Care Systems (ICSs) in England assume statutory responsibility for strategic planning and resource allocation of health and care services.^17^, ^18^ As partnerships between NHS organisations, local government and the voluntary, community and social enterprise sector (VCSE), ICSs are collectively responsible for improving health and reducing health inequalities in their populations.^17^, ^19^ Much larger than CCGs, each ICS has an Integrated Care Board (ICB) responsible for planning and funding most NHS services^17^ and a number of Integrated Care Partnerships (ICPs) which must produce a strategy for health and care services for communities at local ‘place’ and ‘neighbourhood’ level.^20^, ^21^ The principle of subsidiarity necessitates partners to listen and learn together as close to the community as possible ^21^ and embrace the trumpeted organising principle of collaboration for the ‘Long‐Term Plan’^22^, ^23^ The NHS Long‐Term Plan^22^ endeavours to change and improve population health and reduce inequalities, especially where increasing numbers of people have long term conditions and use multiple services.

## 2 SETTING AND METHODS

A focused ethnography was conducted in two case study sites in South‐East England.^24^ This type of rapid ethnography with short‐duration fieldwork is useful for studying healthcare organisation and delivery.^24^, ^25^ The principal research question was: ‘what does it means to work in partnership as clinicians and service users to commission and lead services’.^26^ It encompassed the material conditions and associated bureaucratic processes of commissioning to see if trusted peer relationships were developing for effective PPI. Focused ethnography aims to describe and explain cultural aspects within a group or sub‐group and so uses first‐level questions—the ‘what?’ questions.^27^ Secondary questions probe further and explain; ‘what helps or constrains?’ ^27^

### 2.1 Study design

To obtain contextual background data^28^ three focus groups were initially used, followed by 15 nonparticipant observations at CCG meetings where PPI should have been taking place. Thirteen interviews were subsequently conducted, and relevant documentary sources were analysed. These included minutes of meetings and field notes together with CCG website content.^29^ Material artefacts are important sources of evidence used to confirm or contrast observational and interview data.^27^ Focused ethnographic techniques typically utilise multiple visual, auditory, and photographic artefacts, however, the method was modified to preserve confidentiality and maintain anonymity.

A research project steering committee including three service users and one PPI practitioner, all with commissioning experience in the preceding 5 years, helped assess the rigour of the data collection and interpret the findings, as well as ensure the project remained focused on its intended aims.

### 2.2 Settings and participants

The two case study sites comprised an urban CCG and a rural CCG. Entry to the study sites and access to participants was via the two Engagement Officers responsible for PPI work in their respective CCGs. Participants were therefore a convenience sample. The 11 focus group participants were either exclusively service users and lay representatives or exclusively clinicians (GPs) with a leadership role in the CCG (Table 2). There were eight service users and lay representatives from CCG work streams or GP locality membership meetings, and three GP leads for clinical commissioning. All had participated in PPI practices for commissioning within the two CCGs since April 2013 when the HSCA 2012 was implemented. Data were collected throughout 2016.

**Table 2:** Focus group participants and their clinical commissioning group (CCG) roles.

Focus group	No. of people	Pseudonym and current role in CCG^a^	Additional roles^a^
1. Urban CCG Service users and lay representatives	5	1. Edward—Lay representative on Governing Body (GB) for governance	Deputy chair of GB. Chairs two GB committees. Member of Health & Wellbeing Board
		2. Eddie—Patient Participation Group (PPG) network representative (service user)	Member of PPG
		3. Euan—Lay representative on GB for patient and public engagement	Member of PPG. Co‐chairs Communications and Engagement Committee. Deputy chair GB Quality Assurance Committee.
		4. Elizabeth—Volunteer representative (service user) with Healthwatch on 4 CCG Cancer Groups	Member of PPG
		5. Daniel—Lay representative on Independent Funding Panel	
2. Urban CCG GP Leads	3	1. Simon—GP Lead for Cardio‐vascular disease work stream including stroke prevention	GP Practitioner. Attends PPG in own GP practice.
		2. Ellie—GP Lead for Public Health and Primary Care	GP Practitioner. Working with Local Authority
		3. Zayef—GP Lead–Head of engagement and clinical leadership	GB member
3. Rural CCG Service users	3	6. David—Patient delegate on CCG Quality and Performance Committee (service user)	PPG lead at own GP practice
		7. Hazel—Chair of Patient Representative Group (PRG) north locality and chair of joint PRG locality group meeting (service user)	Member of PPG, member of Programme Board, representative on GP locality group
		8. Hilary—Service user/carer representative on community services procurement	PPG lead at own GP practice

a Job titles and some committee names have been altered.

The 13 interviewees (Table 3) comprised three CCG Governing Body (GB) remunerated lay representatives (for PPI in both CCGs and one for governance), two GP workstream leads, three GP leads for locality GP practice members, two GB GP leads for quality, clinical engagement and leadership and the GP clinical chairs for both CCGs. There was also one service user representative (paid expenses only) involved with service procurement. Three interviewees also took part in the initial focus groups because only two lay representatives were appointed to comply with NHS England CCG requirements.^30^

**Table 3:** Participants by role and gender within both clinical commissioning groups (CCGs).

	Pseudonym	Interview participant	CCG	Gender
1	Euan^a^	GB lay representative for PPI	Urban	Male
2	Hiten	GP locality member Lead	Urban	Male
3	Zayef^a^	GB Lead for engagement and clinical leadership	Urban	Male
4	Edward^a^	GB lay representative for governance	Urban	Male
5	Natalie	GP locality member Lead	Urban	Female
6	Heather	GB Lead for quality	Rural	Female
7	Harriet	Clinical Chair	Rural	Female
8	Lucy	GP Lead for Community Services and GP Lead for Environmental and Social Sustainability	Urban	Female
9	Alison	Accountable Officer and Chief Clinical Officer	Urban	Female
10	Alex	GP Lead for Dementia work stream	Rural	Female
11	Duncan	GP locality member Lead	Rural	Male
12	Leslie	Service user for diabetes service procurement	Rural	Male
13	Nigel	GB lay representative for PPI	Rural	Male

Abbreviation: GB, Governing Body; PPI, patient and public involvement.

a Also took part in a focus group.

### 2.3 Data collection

All data were collected by DH over a period of 12 months. D. H. was a senior lecturer in nursing at the time with experience in involving patients and carers in curriculum design and course delivery. She also had 20 years experience of facilitating a cancer support group where members are involved in research design and service improvement at a local and strategic cancer network level. All participants provided written informed consent. It proved too difficult to conduct a GP lead focus group in the rural CCG due to several factors including the geographical location of rural GP practices. It was also difficult to secure time during the one day a week the GP leads were participating as commissioners on CCG premises.

Each focus group was for approximately 45 min duration. Proceedings were digitally recorded and transcribed by the researcher (D. H.). Both urban CCG focus groups (Table 2) were held on CCG premises. The rural CCG service user focus group was held in the home of one of the service users for participant convenience. The prompt questions (Table 4) were the same for all focus groups.

**Table 4:** Focus group prompt questions.

1. What is ‘working in partnership’ and what does it mean to you? (Definitions) 2. In what ways do patients, carers, and the public work with commissioners? (Examples) 3. What would it mean to trust somebody and feel like a peer to them when discussing commissioning or changes to services? (Values) 4. How would you know if that trusted peer relationship was working or valued? (Feedback and reinforcement)

Observations were completed in the two CCG case study sites and allied documentary sources and material artefacts were collected, totalling 32 pieces of evidence. The observations were made over the course of 11 months at strategic or sub‐committee meetings or work streams where service users and/or public representatives were present. The meetings constituted the regular business of CCGs, and were held every month or 2 months with some required to be conducted in public. Their duration ranged between 75 min and 165 min. It was impractical to observe every PPI practice for clinical commissioning and so those selected provided snapshots for the focused ethnography. The selection was mostly determined by participants referring to meetings they attended and not preplanned because of the iterative nature of the study.

The 13 face‐to‐face interviews were conducted over a period of 8 months between March and November 2016. A semi‐structured interview schedule was used, and all interviews were audio‐recorded, transcribed, and anonymised by a paid transcriber. The transcripts were checked with the participants for accuracy.

### 2.4 Data analysis

**Figure 1:**
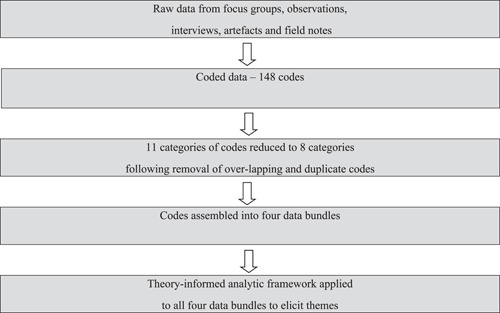
Flow diagram to summarise data management.

The qualitative data analysis software package NVivo version 11 was used to manage and make sense of the large amount of text data. Summaries of ‘data bundles’ are sometimes employed in ethnographic research where there are voluminous data from observations and field notes that need to be condensed.^31^ Figure 1 summarises the data flow and management. Codes and some data bundles were checked by members of the project steering committee and co‐authors KA and BF. The remaining eight categories of codes informed four data bundles of PPI practices for clinical commissioning, designated as trust, leadership, learning and partnership, (Table 5). Two categories, ‘roles’ and ‘Governing Body’ (italicised in Table 5) appear in more than one data bundle.

**Table 5:** Four data bundles from eight categories.

Data bundles	Categories of codes
Trust	Trusting peer to peer
	What success looks like
Leadership	Clinical leadership
	*Governing Body*
	*Roles*
Learning	Development support
	*Roles*
Partnership	Representation
	*Governing Body*
	Partnership working

Analysis was both deductive and inductive. The data bundles were conceived deductively as four sets of situated learning practices (Table 5). A theory‐informed framework was then applied, the components (Table 6) of which were drawn from a range of sociomaterial theories such as social learning theory and practice theories, based on the dynamics of social practice,^32^ professional learning^33^ and ‘communities/landscapes of practice’.^14^, ^34^, ^35^ Both CCGs referred to CoPs as a form of engagement and preferred way of working aligned to good practice. The nature of these is reported elsewhere.^26^ This framework fully attends to the materiality of practices and themes were derived inductively from each set or data bundle, allowing us in this paper to re‐explore and argue for the importance of materials in PPI in commissioning.

**Table 6:** Theoretical framework for data analysis.

Components
Participation—How involvement is engaged, aligned with policy or imagined
Materiality—Concrete or ‘reified’ requirements, e.g. minutes, checklists, guidelines
Competence—Skills, know‐how, knowledge
Boundary encounters—Past, present and CoPs that affect PPI for commissioning
Meanings—The social and symbolic associated with CoPs

### 2.5 Ethics

The study received university ethical approval and underwent proportionate review by the National Research Ethics Service. Letters of Access were also obtained for both CCG settings.

## 3 FINDINGS

We focus on the heavy materiality within CCG engagement and involvement practices that bureaucratise PPI processes, revealing how materiality impacts and can hinder trust, leadership, learning, and partnership working practices. The narrative draws on themes derived inductively from the framework in Table 6. Pseudonyms are used throughout consistent with Tables 2 and 3.

### 3.1 Voluminous governance artefacts

Governing Body (GB), meetings were notable for the large volume of information released to the public 1 week ahead of each meeting. A theme within leadership practices was the multiple ‘artefacts’. The rural CCG released 34 papers before one meeting, and combined papers for another contained 273 pages or nine megabytes of data when downloaded from the CCG website. The urban CCG was no different with around 200 pages in the GB papers collating all the ongoing work including reports from committees and work streams, and financial performance reports. Paper copies were made available, but it was difficult to see how this could be assimilated and followed at the time of the meeting if the content was unfamiliar to the reader.

Lack of time and short lead times for reading large volumes of CCG papers before meetings were identified by the GP leads. CCG work encroached on personal time and was thought to be one reason why it was difficult to recruit GPs to the GB according to the lay representative for governance in the urban CCG.

**Lucy**: There's acres of paperwork, you know, this afternoon we've got the Clinical Strategy Group meeting and … five days before you get sort of a hundred‐page document to read, you know, so yes I do a lot in the evenings and around the edges.

[GP Lead, urban CCG interview]

GB agendas followed a set structure which addressed common features of the meetings including the declaration of interests, minutes of the last meeting and matters arising, governance, quality and performance, delivery and strategy, reports, and minutes from sub‐committees. Most of the content was for noting rather than discussion. The emphasis was on procedure to process and report the work of the CCG rather than decision‐making. The many activities of the CCGs were captured within GB documents using a standardised template for consistency and transparency. Reporting ‘patient and public engagement’ on the template, the CCGs used impersonal phrases such as:

Patient representatives were engaged throughout the procurement exercise and formed part of the evaluation team. They have been consulted on the service model.

[Reporting outcome of procurement for a community service, urban CCG]

### 3.2 Overdominance of centralised agendas

Having to adhere to centralised agendas was often seen as unhelpful and symptomatic of an over‐bureaucratised system. A negative theme from trust practices was ‘process reification and metrics of success’. Meeting procedure did not always engender trust in the measures of success as highlighted by David one of the service users on the CCG's Quality and Performance Committee who attended locality meetings:

**David**: ‘Oh we have finished on time’. ‘Oh, we have finished five minutes early’. ‘Well done, that was a good meeting’. Not what have you achieved?…

So, their whole focus is just about churning the stuff through, producing a report and then passing the report upstairs. It is not about results. It is about process…. I think that is inevitable to some extent. I think it is part of the way bureaucracy works. It is management by numbers…

[Service users focus group, rural CCG]

Natalie, a GP lead in the urban CCG, was equally scathing about the reporting processes to the CCG GB. She felt it was a ‘rubbing stamping’ exercise ‘coming from the centre’ to ‘tick all the boxes’.

**Natalie**: Yes, that is overrated how much we liaise with [GP] practices. In fact, the whole democratic structure is overrated to be honest. It is still coming from the centre and coming out…. we would have locality meetings every two months but really and truly they increasingly become much more rubber stamping. The CCG telling ……General Practice is really pushed. They are too busy and they can't get their heads above water to actually take on all this stuff. Actually, looking back on it they have got all the lovely structures and they can tick all the boxes but in practice ….

[GP locality member Lead, urban CCG interview]

Both Natalie and David used the metaphor of taking/reporting something ‘upstairs’ to the CCG, consistent with Wenger's idea of alignment.^14^ For Natalie though, despite sitting on the GB as the Lead for a GP locality, it felt as if she had no power as a GP commissioner. It was ‘centrally driven’ and she wished she had learnt to be stronger from the outset. With hindsight she said she ‘would be a lot more “ballsy”… with secondary care…with NHS England’. She not only referred to the influence of NHS England, an executive nondepartmental public body overseeing the operationalisation of commissioning, but also the pressures from secondary care which were impacting the work of the GP clinical commissioners:

**Natalie**: We don't have that power. NHS England are still driving it from the centre. You know we are rubber stamping a lot of the time. We don't get that power because you will try and make some decisions and suddenly the decisions are taken upstairs. There has been a letter from NHS England saying this, that and the other. …. Commission acute services effectively and hold them to account. There is always this thing ‘we can't let them fail'. ‘We have got to bail them out’. … ‘the Acute Trust always say if they overspend they will get bailed out’.

[GP locality member Lead, urban CCG interview]

The Chief Clinical Officer (Alison) in the urban CCG, was particularly candid when reflecting on the failing secondary care provider and how this may have impacted on her ability to speak up for the CCG. She spoke of using certain ‘buzzwords’ to ‘keep the minister off your back’. Life for her as a clinician was about complexity and ‘coming at it from a bit more of a disruptive place and I don't think that's done us any favours as a CCG’. She was referring to her two roles as Chief Clinical Officer and Accountable Officer. Separating them into a clinical role and a managerial role with the legal duties would have been her preferred option which is what the CCG opted for at a later stage. She was a clinician at heart, not a leader, and commented on her many years of training to be a doctor and her reluctance to be dragged along a management trajectory.

**Alison: …** I've seen other people, not in as a difficult situation with their acute trust as we're in, so maybe I could not have done it, I might be being hard on myself, but I do think I've seen other leaders manage to get air cover for their commissioning groups by using certain buzzwords, having a phrase, …. it's a couple of sentences probably you need to… and then they get it. Whereas I come at it from a clinician, accepting that the world is complicated, the complexity is what we do, that a buzzword doesn't solve anything. You know, I come from a very different place, whereas what they need is something simple to feed up the line to keep the minister off your back.

[Accountable Officer & Chief Clinical Officer, urban CCG interview]

### 3.3 Insufficient time to build trusted relationships

Time was considered a material resource and impacted trust practices in a variety of ways. Examples included the ‘wasted time’ of sitting through a meeting and no‐one taking comments and contributions seriously (service user focus group), and the inappropriate use of GB time to allow a member of the public to promote a service:

**Natalie**: We shouldn't have had to listen to that at a Governing Body meeting …. That was completely inappropriate use of Governing Body time. …. it stopped other people having other questions … it makes it difficult because he turned up, so he got airtime. How do you get a voice for those people who can't turn up?

[GP locality member Lead, urban CCG interview]

GP leads in both CCGs also spoke of how time‐intensive it was to engage with multiple partners for workstream collaborations. This could be problematic when there were small VCSE organisations contributing to strategic planning and/or involved in procuring and delivering services. There was not enough time or, as the lay representative for PPI in the urban CCG clarified, ‘not enough checking up’ to see if what should have been done had been done.

In other instances, PPI processes took a long time to come to fruition. Soon after taking up the post, the GB lay representative for the urban CCG wanted to change the reporting structures. The participation work was a sub‐committee of a sub‐committee of the Quality Assurance committee:

**Euan**:  … it was like patient and public participation was an afterthought that came on at the end. And I brought it up time and time again to the Governing Body … They just kept on looking at me blankly so in the end I said well, I am not assured that the patient voice is getting into the heart of the CCG. Stuff was happening but it was all below the radar.…

[Lay representative for PPI, urban CCG interview]

It took a year to instigate changes and was not helped by challenges to the lay representative's authority as a leader despite a good rapport and genuineness between meeting participants. The process was resource‐heavy in terms of staff attendance and time and included diarised premeetings.

The precarious nature of trust and the temporal process of trusting relationships was evident early in the study. One of the Governing Body (GB) lay representatives had witnessed staff come and go since the implementation of the new CCG structures in April 2013:

**Euan**: …. without mutual trust and respect, I don't even know if there is any point in having a conversation … how do you develop that trust? Trust builds up over a very long period of time and can be lost (clicks fingers together) like that. …how do you build up trusting relationships with individuals and more collectively in a body where staff are changing? It is very difficult because the CCG is an institution, but it is the people within it that are the institution, and then building up those relationships. …. it does take a lot of time and energy.

[Lay representative for PPI, urban CCG focus group]

### 3.4 Recognition of leadership track record

Consistent with earlier studies of emerging CCGs^36^, ^37^ the findings showed appointments into clinical leadership roles were initially taken up by clinicians (GPs) with prior experience of working in practice‐based commissioning and primary care trusts (PCTs). A recognised leadership track record also applied to the lay representatives familiar with engaging and aligning with NHS agendas. It was a participation theme of leadership practices. Edward spoke of concluding his role with a PCT when the newly formed CCG commandeered him with ‘a little arm twisting’ to join as the lay member for governance. Similarly, the lay representative in the rural CCG took up post before CCG authorisation having impressed as a capable leader with GP leads, GB members and service user representatives alike commenting on his participation.

**Harriet**: … everybody on the Governing Body has done development, you know the PPI Lead, he's been on a lot of development, although he's very self‐driven, he drives a lot of what he does, but he is… we're lucky to have him, he is amazing and he certainly leads by example, …

[Clinical Chair, rural CCG interview].

Competent leadership mattered although the GP Leads appeared to ‘play down’ the roles they occupied in terms of the material aspects of recruitment and selection such as a job description, application form and interview. These were absent and sometimes there was no competitive selection or formalised interview for the clinicians. It was a case of someone having to do the job and willingness to participate based on their experience of leadership and GP clinical engagement. The qualitative and focused nature of the study limits to comment on how widespread this was for recruiting GP leaders in the CCGs at that time. What mattered was GP participation, learning whilst doing, learning as experience and learning as becoming a clinical commissioner.

**Alex**: … I was asked by…Clinical. Chair to come and have a conversation. … And she said well I just want you to go and see what's going on in this area and come back and let me know. … I thought about it and I sent her an email and said well it sounds quite interesting, what do I have to do to apply for it. And she said no, no, you've got it…laughs… the role is yours.

[GP Lead, rural CCG interview]

**Lucy**: I never had a job specification. I asked several times for an induction and I never got one. I asked several times for some sort of specification and the most I've got, from my own research is looking at other Leads’ specifications and seeing….

[GP Lead, urban CCG interview]

Conversely, both GB lay representatives for PPI described detailed material conditions for the recruitment process. Receiving a job description, completing an application form, submitting curriculum vitae (CVs) and being interviewed before the decision to appoint was made. The selection process was rigorous to legitimise their respective positions within their CCG communities yet both individuals had extensive track records in the field of PPI practice. The selection of the lay representative for PPI in the urban CCG (Euan) was particularly onerous with two interview panels conducted on the same day. The first panel was with GB members and the second included voluntary sector and Patient Participation Group (PPG) representatives.

Service users and lay representatives had experiences of engagement and leadership relevant to their CCG roles and leadership practices. However, status was raised early in the study, especially when there was a formal role definition and recruitment process. Some of the service user representatives commented on their lack of status and recognition, a point well‐illustrated in the rural CCG by Hazel when comparing patient representatives to the lay representatives on the GB. They brought a wealth of expertise valued by their peers on the GB, but patients (service users) were only seen as ‘generic’ and were not considered for the skills and expertise they brought from their daily lives. The impact of this lack of material recognition meant that service users did not feel the CCG valued them in the same way as the lay representatives.

**Hazel**: …When they ask us to come along they simply see us as generic patients. Like ‘a person in the street’ but most of us have skills. We have skills in jobs that we have done and they may be skills that they could very much learn from and would help them. And we want to be true partners and give that kind of skill. They haven't understood that we are not sort of just generic patient people.

[Service users, focus group 3, rural CCG]

### 3.5 Incomplete feedback for partnership working

One of the themes for partnership practices was ‘needing feedback’. Clearer feedback that went beyond quantitative, centrally determined key performance indicators was suggested by some participants. Lay representatives and service users wanted to be made known, visible to others, what difference their contributions had made to partnership working. There were frequent references to the need for more feedback. Service users wanted a better understanding of what could and could not be done. This was despite the numerous ways CCG performance was monitored at both local and national levels for NHS England, including annual assessment as part of the NHS Oversight Framework for commissioning regulation.

**Elizabeth**: No‐one actually comes back to you and says the reason we **didn't** [emphasis] do it this way was because of the system, or because we couldn't, or because of funding, or because there just isn't the capacity to do it that way or you know, so you just sort of think well I said that but no one is taking the slightest bit of notice of that and I don't know why.

[Service user representative, focus group 1, urban CCG]

Another process measure criticised was one used for capturing patient experience ‐ Patient Reported Experience Measures.^38^, ^39^ A service user representative at one of the locality meetings was asked to review draft questionnaires to be sent out to patients. She was critical of the content and considered the questions did not measure what was important to patients. It was, however, what the CCG would use to capture data for reporting purposes as part of the monitoring and review of services and no amendments were made.

**Hazel**: … I said at no point did you ever say to a patient ‘What was it you wanted to get out of your treatment and did you get it?’ Which is straight forward. It is a ‘yes’/‘no’. …. It is only when you analyse those sort of issues that you find out whether the process has worked for the patient. Just saying were you able to contact your adviser on a scale of 1 to 5? You either are or you can or you can't.

[Service user representative, focus group 3, rural CCG]

## 4 DISCUSSION

In this paper, we have focused on evidence from an ethnographic study of two CCGs to demonstrate how materiality generates specific bureaucratic forms of PPI for clinical commissioning pre‐dating ICSs. The materiality of public participation and citizen engagement is entangled with objects, reified processes, things or technologies, whilst at the same time this materiality constrains, and elements or ‘things’ go unacknowledged in current public discourse surrounding PPI.^10^ This materialisation of participation challenges the usual deliberative and discursive processes to offer new detailed, specific and contingent accounts of materiality in relation to PPI.^10^ As seen in the work of Wenger^14^ and Wenger‐Trayner^35^ on social learning, and Fenwick^15^, ^33^ on the social and material forces of practice learning, sociomaterial practices reveal the assemblage of materials involved in participation and involvement. This is not just a focus on product or outcome to be measured for impact within communities and work streams for commissioning health and care services. Instead, the technical and methodological aspects of PPI require a deeper understanding of practices.^40^ Using a sociomaterial lens allows us to see how particular forms of PPI, including the PPI subject, emerge, are governed, and how PPI authority or experience is continuously produced or practised, accepted, dismissed and often ignored.

In terms of exploring what it means to work in partnership as clinicians and service users to commission and lead services, the study findings have similarities with earlier research where material conditions impeded rather than enhanced the process. Reasons for not responding to PPI input were often due to the wider systems and how CCGs would be judged on their performance. Centrally‐prescribed evidence from NHS England and national outcomes for the GP contract such as the Quality and Outcomes Framework^41^ took precedence over listening to local input from PPI representatives.^42^ PPI became a lip service as there was no capacity to do more than adhere to target requirements. It was a ‘window‐dressing’ exercise often carried out by managers.^2^ As with this study, recruitment and selection criteria had lay representatives ‘jumping through hoops’^43^ and commissioners defining who has a legitimate voice.^44^ Also, in this study, participation and engagement with service users and public representatives occurred predominantly within the strategic planning stage of the NHS commissioning cycle yet statutory guidance^7^ indicated it should be evident throughout the ‘procuring services’ and ‘monitoring and review’ stages of the cycle. The GP leads appeared to engage with service users at the planning stage and then the later stages, and the processes therein, were completed and reported by the commissioning support team. Service user representatives did not always know the outcome of the commissioning cycle.

The PPI experiences of CCGs can provide useful lessons if clinicians, service users and the public can work together in a trusted peer relationship. There is an opportunity to move beyond the ‘atheoretical way of getting input’^45^ for PPI in clinical commissioning which Hazel criticised in her reference to the objective data for Patient Reported Experience Measures. The ICPs will require time to embrace and fully embed collaborative processes if things are to be done differently to improve population health and reduce inequalities for the NHS Long‐Term Plan.^22^ It is, therefore, crucial that PPI voices are not lost in further bureaucracy which might come with scaling up despite intentions to the contrary. It remains a legal requirement to involve and engage service users and the public in the planning and commissioning of services.^46^ Early findings from NHS Sustainability and Transformation Partnerships, a strategic precursor of ICSs, charged with building networks across the local health economy and breaking down organisational and hierarchical barriers^20^, ^46^, ^47^ indicate public accountability is lacking.^48^ Preoccupation with development of system service provider collaboratives has been a factor.^48^ Recent interviews with 25 chief executives and chairs of ICBs and ICPs suggest concerns remain about ICS central command and control when more freedom and flexibility should be devolved to place‐based partnerships and local neighbourhoods involving people and communities.^20^

Despite limited space to discuss here, attention is drawn to the ideas of Lipsky^49^ and street‐level bureaucracies where public sector workers directly influence citizens’ experience of government policy. Although described some 40 years ago in terms of exercising discretion and control over clients, there are parallels with the current health and care landscape in terms of fiscal constraints and performance management overseen by NHS England.^50^ Are citizens in places and neighbourhoods in danger of having their needs constructed, as Lipsky^49^ suggests, by way of the burdensome materiality of commissioning? Dimensions of control included the benefits and sanctions of service delivery by various agencies, structuring the context of interactions, teaching how clients should behave and assigning psychological rewards and sanctions with respect to the relationships formed with the street‐level bureaucrats.^49^ To some degree these aspects were evident in the study data although the new breed of nonclinical commissioners was a welcome departure.

A limitation of the study is that the work of the commissioning support managers was not captured more fully within the focused ethnography. One GP lead referred to her ‘awe‐inspiring’ commissioner, but comments came from all study participants. The commissioners, new to post in both CCGs, were influencing the service users and lay representatives as well as the clinicians. They brokered and led some of the negotiations in the various work streams or communities of practice. They were a new type of commissioner with a different approach and skill set for learning together and in partnership. Reliable and speaking on the same level without effect or superiority were two features of their practice that instilled trust.

## 5 CONCLUSION

Although findings from qualitative research cannot be generalised, this sociological‐focused ethnography allowed a pragmatic approach to exploring PPI social practices for clinical commissioning. In this paper we have explored the materiality affecting and frequently hindering commissioning processes due to excess bureaucracy relative to trust, leadership, learning, and partnership working. Real‐time ‘snapshots’ of practice provided a deeper examination of PPI activity for clinical commissioning. It helped articulate the visible and hidden practices shaping PPI in the two CCGs and suggests much closer attention must be paid to the explicit and tacit social meanings and materialities of partnership. An example of ‘hidden’ practices being the excessive recruitment processes of the GB lay representative for PPI in the urban CCG.

System leaders in the new ICSs should reconsider the significance of the material conditions from centrally driven PPI processes and lessen the negative effects if true partnerships are to be achieved within place‐based communities and neighbourhoods.

## AUTHOR CONTRIBUTIONS

**Debbie Hatfield**: Conceptualisation (co‐lead); writing—original draft (lead); investigation; methodology (lead); formal analysis (lead); writing—review and editing (equal). **Kay Aranda**: Supervision; conceptualisation (co‐lead); writing—original draft (supporting); methodology (supporting); formal analysis (supporting); writing—review and editing (lead). **Gordon Ferns**: Funding acquisition; supervision (lead); project administration; resources; writing—review and editing (supporting). **Breda Flaherty**: Funding acquisition; supervision; project administration; methodology (supporting); formal analysis (supporting) writing—review and editing (supporting). **Angie Hart**: Conceptualisation (supporting); writing—review and editing (equal).

## CONFLICT OF INTEREST STATEMENT

The authors declare no conflict of interest.

## ETHICS STATEMENT

The study received ethical approval from Brighton and Sussex Medical School Research Governance and Ethics Committee—Ref No. 15/080/FER. The National Research Ethics Service (NRES) under the auspices of the Health Research Authority gave a favourable opinion by proportionate review—Ref No. 15/SW/0214. Local research and development ethical approval was granted by the two CCGs and Letters of Access were obtained for both settings. All participants provided informed written consent.

## Data Availability

The data that support the findings of this study are available on request from the corresponding author. The data are not publicly available due to privacy or ethical restrictions.
